# Inhibition of circular JUN prevents the proliferation and invasion of glioblastoma via miR‐3064‐IGFBP5 axis

**DOI:** 10.1111/jcmm.70098

**Published:** 2024-09-22

**Authors:** Yuhao Zhang, Shiming Liu, Cheng Wu, Xin Gao, Hongtao Zhao, Ou Li, Faliang Gao

**Affiliations:** ^1^ Cancer Center, Department of Neurosurgery, Zhejiang Provincial People's Hospital (Affiliated People's Hospital) Hangzhou Medical College Hangzhou Zhejiang China; ^2^ General Surgery, Cancer Center, Department of Gastrointestinal and Pancreatic Surgery, Zhejiang Provincial People's Hospital (Affiliated People's Hospital) Hangzhou Medical College Hangzhou Zhejiang China

**Keywords:** circJUN, glioblastoma, IGFBP5, miR‐3064

## Abstract

Glioblastoma (GBM) remains one of the most aggressive and lethal brain tumours, characterized by rapid progression and limited treatment options. This study investigated the regulatory roles of circular RNA circJUN, and its functional interaction with microRNA miR‐3064 in GBM pathogenesis. We employed bioinformatic analyses and clinical sample validation to identify circJUN as a potential target in GBM. Subsequently, we engineered GBM cell lines with stable circJUN knockout or overexpression, and transfected them with miR‐3064 mimic/inhibitor or IGFBP5 small interfering RNA (siRNA)/expression vector to elucidate the molecular mechanisms governing GBM proliferation and invasion. To investigate the in vivo effects, xenograft tumour models were established in nude mice using engineered cells to assess the roles of circJUN in tumour growth regulation. Our analyses revealed significant overexpression of circJUN in GBM tissues compared to healthy controls, which strongly correlated with poor patient prognosis. In vitro and in vivo experiments demonstrated that circJUN overexpression could enhance GBM cell proliferation and invasion. Mechanistic investigations uncovered EIF4A3 as an interacting factor of circJUN which promotes circJUN expression, and circJUN modulates miR‐3064 activity to regulate the malignancy of GBM cells. Furthermore, we identified IGFBP5, a crucial regulator of cell growth, as a direct target of miR‐3064, thereby establishing an additional layer of control over GBM proliferation and invasion. Our study unveils a complex regulatory network involving circJUN, miR‐3064 and IGFBP5 in GBM pathogenesis, underscoring their potential as novel therapeutic targets for improving patient outcomes. Our findings not only contribute to the understanding of GBM biology but also pave the way for innovative therapeutic approaches in the management of this malignancy.

## 1 INTRODUCTION

Glioblastoma (GBM) stands as one of the most aggressive and lethal brain tumours, characterized by rapid growth and invasive behaviour, with limited efficacy of conventional therapies.^1^, ^2^ Despite advancements in treatment modalities, including surgical resection, radiotherapy and chemotherapy, the prognosis for GBM patients remains dismal, compounded by challenges in early detection.^3^ The highly invasive nature of GBM, coupled with its ability to evade therapeutic interventions, underscores the urgent need for novel treatment strategies to improve long‐term survival outcomes.^3^ Current statistics paint a grim picture, with median survival duration post‐diagnosis hovering at a mere 15–18 months.^4^ Elucidating the molecular mechanisms underlying GBM pathogenesis is therefore crucial for developing effective therapies that can significantly enhance both survival rates and quality of life for GBM patients.

Circular RNAs (circRNAs) represent a novel class of non‐coding RNAs characterized by a unique single‐stranded, covalently closed structure formed by the joining of 3′ and 5′ ends.^5^ These molecules have emerged as critical regulators in various physiological and pathological processes, with particularly significant roles in oncology.^5^ CircRNAs exhibit diverse functionalities, acting as either tumour suppressors or promoters by modulating cellular proliferation, apoptosis, invasion and inflammation.^5^ Recent studies have illuminated the involvement of specific circRNAs in GBM pathogenesis. For instance, circPARP4 has been implicated in GBM progression, significantly enhancing proliferation and invasion capabilities through regulation of miR‐125 expression.^6^ Similarly, circMMP9 has been shown to potentiate migration and invasion of GBM cells via the miR‐124/EIF4A3 axis.^7^ Elucidating the functional roles of circRNAs in GBM holds promise for identifying novel molecular targets and developing therapeutic strategies aimed at inhibiting GBM invasion and proliferation.

MicroRNAs (miRNAs) are small non‐coding RNA molecules that play crucial roles in regulating protein expression, either by inducing mRNA degradation or inhibiting their protein translation. Aberrant expression of miRNAs has been implicated in various aspects of GBM pathogenesis, including tumour growth, invasion, angiogenesis and therapeutic resistance.^8^ These miRNAs exhibit diverse functionalities in GBM, acting as either oncogenes or tumour suppressors. Several miRNAs have been identified as upregulated in GBM, functioning as tumour promoters. Notable examples include miR‐21, miR‐93 and miR‐10b.^9^, ^10^ Conversely, miRNAs such as miR‐34a, miR‐137 and miR‐146a have been found to be downregulated in GBM, suggesting their potential roles as tumour suppressors.^11^, ^12^, ^13^ The impact of miRNAs on tumour cell malignancy is largely determined by their downstream protein targets and the associated biological processes. Elucidating the intricate interplay is crucial for unravelling the complex mechanisms underlying GBM development and progression.

In this study, we identified circJUN as a heavily upregulated circRNA in GBM, and investigated its functional role and the underlying molecular mechanism in dictating the malignancy of GBM cells. We validated the overexpression of circJUN in clinical GBM samples and assessed its correlation with patient outcomes. A series of in vitro and in vivo experiments were conducted, including stable circJUN knockout and overexpression in GBM cell lines, to assess its effects on cell proliferation and invasion. We further explored the interactions between EIF4A3, circJUN, and miR‐3064. Additionally, we investigated the downstream target of miR‐3064, identifying IGFBP5 as a key player in this regulatory network. Our findings reveal a novel circJUN/miR‐3064/IGFBP5 axis in GBM pathogenesis, providing new insights into the complex molecular landscape of this aggressive brain tumour. This study not only enhances our understanding of GBM biology but also identifies potential therapeutic targets for improving patient outcomes in this challenging malignancy.

## 2 METHODS

### 2.1 Tissue specimens

This study enrolled 68 patients diagnosed with GBM according to the 2021 World Health Organization (WHO) Classification of Tumours of the Central Nervous System.^14^ All patients were diagnosed and treated at Zhejiang Provincial People's Hospital between January 2018 and December 2022. Inclusion criteria mandated that patients had not received any prior chemo‐ or radiotherapy. GBM tissue samples were obtained during initial surgical resection procedures. For the control group, we collected normal brain tissue samples from 20 patients undergoing brain tissue resection due to non‐neoplastic conditions, primarily traumatic brain injuries, at the same institution during the same period. All tissue samples were collected under sterile conditions in the operating room. Immediately after excision, samples were flash‐frozen in liquid nitrogen and stored at −80°C for subsequent RNA and protein analyses. The study protocol was reviewed and approved by the Medical Ethics Committee of Zhejiang Provincial People's Hospital. Written informed consent was obtained from all participants or their legal representatives before sample collection and data usage.

### 2.2 GBM cell culture, transfection and stable cell line generation

Human GBM cell lines (A172, U251, T98, LN229) and normal astrocytes were obtained from the American Type Culture Collection (ATCC, Manassas, VA, USA). All cell lines were cultured in Dulbecco's Modified Eagle Medium (DMEM; Gibco, Thermo Fisher Scientific, Waltham, MA, USA) supplemented with 10% foetal bovine serum (FBS; Gibco) and 1% Penicillin–Streptomycin (Gibco) at 37°C in a humidified incubator with 5% CO_2_.

For stable cell line generation, lentiviral vectors pLKO.1‐sh‐NC (negative control), pLKO.1‐sh‐circJUN, pCDH‐CMV‐MCS‐EF1‐Puro (empty vector) and pCDH‐CMV‐circJUN‐EF1‐Puro were constructed by KeyGEN BioTECH (Nanjing, Jiangsu, China). To generate the circJUN construct, the linear circJUN sequence flanked with intronic sequences that promote back‐splicing was PCR‐amplified from human glioma genomic DNA and cloned into the pCDH‐CMV‐MCS‐EF1‐Puro vector. Finally, the construct was verified through sequencing to ensure correct insertion and orientation. For circJUN knockdown, the following shRNA sequences were cloned into the pLKO.1 vector between the AgeI and EcoRI restriction sites: sh‐NC (scramble control): GCGTGACGAGCTCGAGGCCGC‐3′; sh‐circJUN#1: 5′‐GGTGCCGCGCGCGAGTCGACA‐3′; sh‐circJUN#2: 5′‐CGGGTGCCGCGCGCGAGTCGA‐3′; and sh‐circJUN#3: 5′‐GCCAGCGGGTGCCGCGCGCGA‐3′. Lentiviral particles were produced in HEK293T cells using the Mission Lentiviral Packaging Mix (Sigma‐Aldrich, St. Louis, MO, USA) according to the manufacturer's protocol. Briefly, HEK293T cells were co‐transfected with the lentiviral vector of interest and the packaging mix using Lipofectamine 3000 (Thermo Fisher Scientific, Waltham, MA, USA). Viral supernatants were collected 48 and 72 h post‐transfection, filtered through a 0.45‐μm filter, and used to transduce GBM cells. For transduction, GBM cells were seeded in 6‐well plates at a density of 2 × 10^5^ cells per well and incubated overnight to achieve approximately 70% confluency. The filtered lentiviral supernatant was added to the cells at a multiplicity of infection (MOI) of 10, supplemented with 8 μg/mL polybrene (Sigma‐Aldrich) to enhance transduction efficiency. After 24 h, the medium was replaced with fresh complete medium. Successfully transduced cells were selected using puromycin (2 μg/mL; Sigma‐Aldrich) for 10 days, beginning 48 h post‐transduction, to generate cells stably expressing shRNA or circJUN sequence.

For transient transfections, the full‐length open reading frames (ORFs) of EIF4A3 and IGFBP5 were PCR‐amplified from human glioma cDNA and cloned into the pcDNA3.1 vector to generate pcDNA3.1‐EIF4A3 (ORF‐EIF4A3) and pcDNA3.1‐IGFBP5 (ORF‐IGFBP5), respectively. The shRNA targeting EIF4A3 was designed and cloned into the pSilencer vector (pSilencer‐sh‐EIF4A3) with the sequence: 5′‐GGTTTCGGGGATAGACTCCTGTAG‐3′. Synthetic siRNAs were used for IGFBP5 knockdown (si‐IGFBP5) and as a negative control (si‐NC). The si‐IGFBP5 sequence was 5′‐ACCGCGGCAAGCCATCAATCCACT‐3′, while si‐NC sequence was 5′‐GATCAACTCACGACCTCGCGACAC‐3′. The above plasmids and siRNAs were produced by KeyGEN BioTECH (Nanjing, Jiangsu, China). These plasmids and siRNAs were transfected into GBM cells using Lipofectamine 3000 (Thermo Fisher Scientific, Waltham, MA, USA) according to the manufacturer's instructions. Cells were seeded in 6‐well plates at 2 × 10^5^ cells per well and transfected at 70% confluency. For plasmid transfections, 2.5 μg of DNA was used per well, while siRNAs were transfected at a final concentration of 50 nM. The transfection medium was replaced with fresh complete medium after 6 h, and cells were harvested for subsequent experiments 48 h post‐transfection.

For miRNA manipulation, miR‐3064 mimic (a synthetic double‐stranded RNA oligonucleotide designed to mimic the endogenous miR‐3064) and miR‐3064 antagomir/inhibitor (a chemically modified, single‐stranded RNA analog complementary to miR‐3064, designed to inhibit its function) were used to modulate miR‐3064 levels. miR‐3064 mimic, inhibitor and their corresponding negative controls (mimic‐NC and antagomir‐NC) were synthesized by RiboBio (Guangzhou, Guangdong, China). Transfection was performed using Lipofectamine 3000 reagent (Thermo Fisher Scientific, Waltham, MA, USA). Briefly, 100 nM of miRNA mimic, antagomir, or their respective controls were complexed with Lipofectamine 3000 in Opti‐MEM medium (Gibco) for 20 min at room temperature before being added to the cells. The transfection medium was replaced with fresh complete medium after 6 h, and cells were harvested for subsequent experiments 48 h post‐transfection.

### 2.3 RNA isolation, treatment and qRT‐PCR

Total RNA was extracted using the RNeasy Mini Kit (Qiagen, Hilden, Germany) following the manufacturer's instructions. To assess the stability of circJUN, RNA samples were treated with RNase R (KeyGEN BioTECH, Nanjing, China) at a concentration of 3 U/μg at 37°C for 15 min. For the actinomycin D treatment stability assay, cells were treated with 2 μg/mL actinomycin D (Sigma‐Aldrich, St. Louis, MO, USA) to inhibit transcription, and RNA was extracted at 0, 4, 8, 12, and 24 h post‐treatment. The harvested RNA samples were directly used for quantitative reverse transcription PCR (qRT‐PCR) analysis. For miRNA analysis, reverse transcription was performed using the TaqMan MicroRNA Reverse Transcription Kit (Thermo Fisher Scientific). cDNA synthesis for mRNA and circRNA, and subsequent qRT‐PCR were performed using the SuperScript IV One‐Step RT‐PCR System and TaqMan Fast Advanced Master Mix (Thermo Fisher Scientific). All qPCR reactions were conducted on a QuantStudio 5 Real‐Time PCR System (Thermo Fisher Scientific). GAPDH served as the endogenous control for cytoplasmic RNA, while U6 was used for nuclear RNA and miRNA normalization. Relative gene expression was calculated using the 2^−ΔΔCt^ method. The primer sequences were as follows: circJUN forward: 5′‐GAGCGAGCTGGTGAGGAG‐3′, reverse: 5′‐GCGCAGGGTTAATTAAGATGC‐3′; hsa‐miR‐3064‐5p forward: 5′‐CGCGTCTGGCTGTTGTGGT‐3′, reverse: 5′‐AGTGCAGGGTCCGAGGTATT‐3′; IGFBP5 forward: 5′‐ACCTGAGATGAGACAGGAGTC‐3′, reverse: 5’‐GTAGAATCCTTTGCGGTCACAA‐3′; GAPDH forward: 5′‐GGAGCGAGATCCCTCCAAAAT‐3′, reverse: 5’‐GGCTGTTGTCATACTTCTCATGG‐3′; U6 forward: 5′‐GCTTCGGCAGCACATATACTAAAAT‐3′, reverse: 5’‐CGCTTCACGAATTTGCGTGTCAT‐3′.

### 2.4 Western blot

Cells were lysed in RIPA buffer supplemented with protease inhibitor cocktail (Sigma‐Aldrich). Protein concentrations were determined using the Pierce BCA Protein Assay Kit (Thermo Fisher Scientific). Twenty micrograms of total protein per lane were separated by 10% sodium dodecyl sulphate–polyacrylamide gel electrophoresis (SDS–PAGE). Proteins were then transferred to nitrocellulose membranes and blocked with 5% BSA in TBST for 1 h at room temperature. The membranes were incubated overnight at 4°C with primary antibodies: anti‐EIF4A3 (1:1000, Cat# ab32485, Abcam, Cambridge, UK), anti‐IGFBP5 (1:1000, Cat# ab4255, Abcam), and anti‐GAPDH (1:1000, Cat# ab8245, Abcam). The following day, membranes were washed with TBST and incubated with HRP‐conjugated secondary antibody (1:5000, goat anti‐rabbit IgG, Cat# 1706515, Bio‐Rad, Hercules, CA, USA) for 1 h at room temperature. Protein bands were visualized using Pierce ECL Western Blotting Substrate (Thermo Fisher Scientific) and imaged using a Bio‐Rad ChemiDoc MP Imaging System. Band intensities were quantified using ImageJ software (National Institutes of Health, Bethesda, MD, USA).

### 2.5 Fluorescence in situ hybridization (FISH)

For FISH analysis of circJUN, U251 and LN229 cells were seeded at a density of 5 × 10^5^ cells per well in 6‐well plates containing sterile coverslips and incubated overnight. Cells were then washed twice with ice‐cold PBS and fixed with 4% paraformaldehyde for 15 min at room temperature. After fixation, cells were permeabilized with 0.5% Triton X‐100 in PBS for 10 min at 4°C. The Cy3‐labelled circJUN probe (RiboBio, Guangzhou, China) was hybridized to the cells in hybridization buffer (50% formamide, 5× SSC, 500 μg/mL yeast tRNA, 10% dextran sulphate) at 37°C overnight in a humidified chamber. The next day, cells were washed three times with 2× SSC at 42°C to remove excess probe. Nuclei were counterstained with DAPI (1 μg/mL) for 5 min at room temperature. Coverslips were mounted on glass slides using ProLong Gold Antifade Mountant (Thermo Fisher Scientific, Waltham, MA, USA). Fluorescence images were acquired using a Zeiss LSM 880 confocal microscope (Carl Zeiss, Oberkochen, Germany).

### 2.6 Immunohistochemistry (IHC) staining

IHC was performed on formalin‐fixed, paraffin‐embedded (FFPE) tumour tissue sections. The sections were subjected to deparaffinization in xylene and rehydration through a graded ethanol series. Antigen retrieval was performed by incubating slides in 0.01 M sodium citrate buffer (pH 6.0) at 95°C for 30 min. Endogenous peroxidase activity was quenched with 3% hydrogen peroxide for 10 min. Slides were then blocked with 10% normal horse serum (Gibco, Waltham, MA, USA) in PBS for 1 h at room temperature. Primary antibody incubation was performed overnight at 4°C using anti‐IGFBP5 antibody (1:200, Cat# ab216622, Abcam, Cambridge, UK) and anti‐Ki‐67 antibody (1:100, Cat# ab15580, Abcam). After washing three times with TBS‐T (TBS + 0.1% Tween‐20), slides were incubated with HRP‐conjugated secondary antibody (1:500, goat anti‐rabbit IgG, Cat# ab6721, Abcam) for 1 h at room temperature. Protein expression was visualized using the SignalStain DAB Substrate Kit (Cat# 8059, Cell Signaling Technology, Danvers, MA, USA). Slides were counterstained with haematoxylin, dehydrated through graded ethanol and xylene and mounted with Permount (Fisher Scientific, Hampton, NH, USA). Images were acquired using an Olympus BX53 microscope equipped with a DP80 camera (Olympus, Tokyo, Japan).

### 2.7 Luciferase reporter assays

Wild‐type (WT) and mutant (MUT) binding sequences of circJUN or IGFBP5 mRNA 3′UTR containing the predicted miR‐3064 binding sites were synthesized and cloned into the pmirGLO Dual‐Luciferase miRNA Target Expression Vector (Cat# E1330, Promega, Madison, WI, USA). U251 and LN229 cells were seeded in 24‐well plates at a density of 5 × 10^4^ cells per well and cultured overnight. Cells were then co‐transfected with 500 ng of the constructed pmirGLO plasmids (WT or MUT) and 50 nM miR‐3064 mimic or negative control mimic using Lipofectamine 3000 reagent. After 48 h of transfection, luciferase activities were measured using the Dual‐Luciferase Reporter Assay System (Cat# E1910, Promega) following the manufacturer's protocol. Luminescence was detected using a Centro LB960 XS3 microplate luminometer (Berthold Technologies, Bad Wildbad, Germany). Firefly luciferase activity was normalized to Renilla luciferase activity, and the results were expressed as relative luciferase activity (Firefly/Renilla).

### 2.8 RNA pulldown assay

Biotinylated circJUN probes and a control probe with scrambled sequence were synthesized by Biomics Biotechnology (Beijing, China). The circJUN probe was a synthetic biotinylated circular RNA corresponding to the full circJUN sequence, while the control probe was a biotinylated circular RNA of similar length with a scrambled sequence. U251 and LN229 cells were grown to 80%–90% confluency in 15 cm dishes, washed with ice‐cold PBS, and lysed in RIP lysis buffer (20 mM Tris–HCl pH 7.5, 150 mM NaCl, 1 mM EDTA, 0.5% NP‐40, 10% glycerol) supplemented with protease inhibitor cocktail and RNase inhibitor. Cell lysates were sonicated on ice and centrifuged at 14,000 × *g* for 15 min at 4°C to remove cellular debris. The cleared lysates were incubated with 3 μg of biotinylated circJUN probe or control probe overnight at 4°C with gentle rotation. Subsequently, 50 μL of Dynabeads MyOne Streptavidin C1 magnetic beads (Thermo Fisher Scientific) were added to each sample and incubated for 4 h at 4°C with gentle rotation. The bead‐RNA complexes were washed five times with RIP wash buffer (50 mM Tris–HCl pH 7.5, 150 mM NaCl, 1 mM EDTA, 0.1% NP‐40). RNA was extracted from the bead‐RNA complexes using TRIzol reagent (Thermo Fisher Scientific) followed by purification with the RNeasy Mini Kit (Qiagen, Hilden, Germany). The pulled‐down RNA was then analysed by qRT‐PCR to detect the enrichment of miR‐3064. For protein analysis, the protein content in the bead‐RNA complexes was eluted using 2X Laemmli buffer (Sigma‐Aldrich), and the protein samples were subjected to Western blot analysis.

### 2.9 RNA immunoprecipitation (RIP)‐qPCR

U251 and LN229 cells were lysed in RIP lysis buffer supplemented with protease and RNase inhibitors. Lysates were incubated with magnetic beads conjugated to anti‐EIF4A3 (1:1000, Cat# ab32485, Abcam), anti‐AGO2 antibody (Cat# 03‐110, Millipore) or normal mouse IgG (Cat# 12‐371, Millipore) as a control, using the Magna RIP Kit (Cat# 17‐700, Millipore). After washing, RNA‐protein complexes were treated with proteinase K. RNA was extracted using TRIzol and purified with the RNeasy Mini Kit (Qiagen). Reverse transcription and qPCR were performed to quantify circJUN and miR‐3064, with the input sample as a control. Relative levels of circJUN and miR‐3064 in AGO2‐RIP versus IgG‐RIP was calculated using the 2^‐ΔΔCt method.

### 2.10 Cell proliferation measurement

Cell proliferation was assessed using the CCK‐8 assay (Cat# KGA317, KeyGen Biotech, Nanjing, China) and EdU incorporation assay (Cat# C0071S, Beyotime, Shanghai, China). For CCK‐8, cells were seeded at 3 × 10^3^ cells/well in 96‐well plates. After treatment/transfection, 10 μL of CCK‐8 solution was added to each well and incubated for 2 h at 37°C. Absorbance at 450 nm was measured using an RT‐6000 microplate reader (Rayto, Shenzhen, China). For EdU assay, cells were seeded as above and incubated with 10 μM EdU for 2 h at 37°C. Cells were then fixed with 4% paraformaldehyde, permeabilized and stained according to the manufacturer's protocol. Nuclei were counterstained with DAPI. EdU‐positive cells were visualized and counted using a Zeiss LSM 880 confocal microscope.

### 2.11 Migration and invasion assays

Cell migration was assessed using Transwell chambers (8 μm pore size, Corning, NY, USA) without Matrigel coating. Cells (3 × 10^4^) were seeded in the upper chamber in serum‐free DMEM, with 10% FBS‐containing DMEM in the lower chamber as a chemoattractant. After 24 h of incubation at 37°C, non‐migrated cells on the upper surface were gently removed with a cotton swab. Migrated cells on the lower surface were fixed with 4% paraformaldehyde and stained with 0.1% crystal violet. For invasion assays, Transwell chambers were pre‐coated with Matrigel (Corning) to form a reconstituted basement membrane. The same number of cells was seeded in serum‐free DMEM in the upper chamber, with 10% FBS‐containing DMEM in the lower chamber. After 24 h, non‐invading cells were removed, and invaded cells were fixed and stained as described for the migration assay. For both assays, cells were counted in five random fields per insert under a light microscope (200× magnification).

### 2.12 In vivo tumour formation assay

BALB/c nude mice (male, 4–6 weeks old, *n* = 5 per group) were obtained from Shanghai SLAC Laboratory Animal Co., Ltd. (Shanghai, China) and acclimated for 2 weeks in the animal research centre under a 12/12‐h light/dark cycle with ad libitum access to food and water. U251 stably expressing sh‐circJUN, LN229 cells with circJUN overexpression construct, and the corresponding control cells were established using lentiviral transduction and puromycin selection as previously described in cell culture method. Subcutaneous xenografts were established by injecting 1 × 10^7^ U251 cells or 1 × 10^6^ LN229 cells from different groups into the right flank of each mouse. Tumour volume was measured every 7 days for 35 days using digital callipers and calculated as (length × width^2^)/2. On day 35, mice were euthanized by CO_2_ inhalation followed by cervical dislocation. Tumours were excised, weighed and either snap‐frozen in liquid nitrogen for gene expression analysis or fixed in 10% neutral buffered formalin for histological examination. All animal procedures were approved by the Animal Use and Care Committee of Zhejiang Provincial People's Hospital and conducted in accordance with institutional guidelines for animal welfare.

### 2.13 Statistical analysis

All data are presented as mean ± standard deviation (SD) from at least three independent in vitro experiments or from different individual clinical or animal samples as indicated. Statistical analyses were performed using GraphPad Prism software (version 10.0, GraphPad Software, San Diego, CA, USA). Significant differences were determined by two‐tailed unpaired Student's *t*‐test for two groups or one‐way ANOVA followed by Tukey's post hoc test for multiple comparisons. A *p*‐value <0.05 was considered statistically significant.

## 3 RESULTS

### 3.1 CircJUN is overexpressed in GBM tissues and correlates with patient survival

**FIGURE 1:**
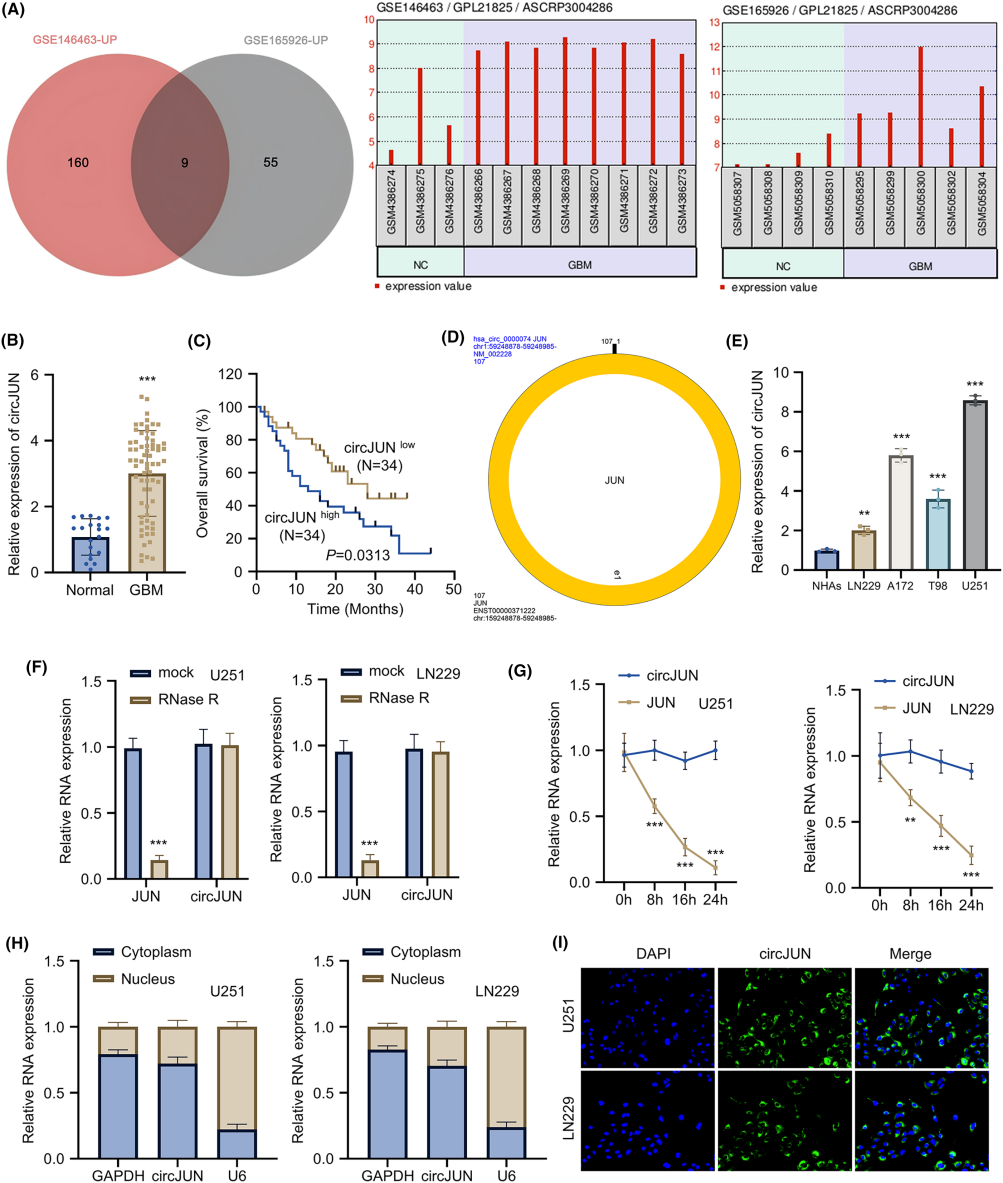
CircJUN is upregulated in glioblastoma (GBM) tissues and correlates with poor prognosis. (A) Venn diagram showing overlapping upregulated circRNAs in GBM from GSE165926 and GSE146463 datasets. Analysis performed using EdgeR (*p* < 0.01, fold change >4). (B) Expression levels of circJUN in 68 GBM tissues and 20 normal brain tissues, as determined by qRT‐PCR. (C) Kaplan–Meier survival analysis of GBM patients with high (*n* = 34) and low (*n* = 34) circJUN expression. The median expression level was used as the cutoff. (D) Schematic representation of circJUN formation and the back‐splicing site. (E) Relative expression of circJUN in GBM cell lines (A172, U251, T98, LN229) and normal astrocytes, as measured by qRT‐PCR. (F) RNase R treatment assay showing the stability of circJUN compared to linear JUN mRNA in U251 and LN229 cells. (G) Actinomycin D treatment assay demonstrating the half‐life of circJUN and linear JUN mRNA in U251 and LN229 cells. (H) Nuclear‐cytoplasmic fractionation followed by qRT‐PCR showing the subcellular localization of circJUN in U251 and LN229 cells. GAPDH and U6 were used as cytoplasmic and nuclear controls, respectively. (I) Fluorescence in situ hybridization (FISH) analysis of circJUN localization in U251 and LN229 cells. CircJUN probe (Green), and nuclei (blue) (*n* = 3 unless especially mentioned in figure panel (*n* = 68/20 for panel B, and *n* = 34 for panel C), **p < 0.01, ***p < 0.001).

To profile differentially expressed circRNAs in GBM, we analysed publicly available datasets from the Gene Expression Omnibus (GEO) database. Analysis of GSE165926 dataset revealed that 64 circRNAs were highly expressed in GBM compared to controls, while GSE146463 showed 1694 upregulated circRNAs in GBM samples. Among the nine common circRNAs between these datasets, hsa_circ_0000074 (circJUN) was selected for further investigation in GBM (Figure 1A). To verify the change of circJUN in GBM tissues, GBM tumour tissues were collected from 68 GBM patients, while normal brain tissue samples were obtained from 20 patients undergoing brain tissue resection due to traumatic brain injuries. qRT‐PCR analysis confirmed significantly higher expression of circJUN in GBM tissues compared to the control specimens (Figure 1B). The GBM patients were categorized into low‐ and high‐expression groups using the median circJUN expression value as the cutoff. Importantly, circJUN expression strongly correlated with GBM patient mortality, with higher expression associated with lower 40‐month survival rates (Figure 1C). Clinical correlation analysis revealed that circJUN expression was significantly associated with the presence of necrosis on MRI, while other factors showed no significant correlation (Table 1).

**TABLE 1:** Correlation of the expression of circJUN with clinicopathologic features in GBM patients.

Parameters	*N* = 68	circJUN expression		
		High *N* = 34	Low *N* = 34	*p*‐value
Age, years				0.625
<60	30	14	16	
≥60	38	20	18	
Gender				0.223
Male	31	18	13	
Female	37	16	21	
Karnofsky performance status scale				0.139
<60	28	17	11	
≥60	40	17	23	
Mean tumour diameter, cm				0.3
<5	22	9	13	
≥5	46	25	21	
Presence of necrosis on MRI				0.014
Yes	40	15	25	
No	28	19	9	
Seizure				0.22
Yes	29	12	17	
No	39	22	17	
WHO grade				0.028
I–II	31	20	11	
III–IV	37	14	23	

CircJUN is formed by the circularization of exon 1 of the precursor JUN mRNA, with a length of 107 nucleotides (Figure 1D). CircJUN expression was significantly elevated in GBM cell lines (U251, A172, T98, and LN229) compared to Normal Human Astrocytes (NHAs). The expression levels followed a descending order of U251 > A172 > T98 > LN229 > NHAs, with all GBM cell lines showing statistically significant higher expression than NHAs (Figure 1E). In both U251 and LN229 cells, this circular structure of circJUN conferred resistance to RNase degradation when compared to the linear JUN mRNA (Figure 1F). Stability assays further demonstrated that circJUN levels remained constant over 24 h after actinomycin D treatment, while the levels of linear JUN mRNA were heavily reduced in both cell lines after transcription inhibition (Figure 1G). Subcellular localization studies using qRT‐PCR on fractionated RNA samples showed that circJUN was predominantly located in the cytoplasm, with limited nuclear expression (Figure 1H). FISH analysis further confirmed the cytoplasmic localization of circJUN in both LN229 and U251 cell lines (Figure 1I).

### 3.2 Modulating circJUN expression level affects the proliferation and invasion of GBM cells

**FIGURE 2:**
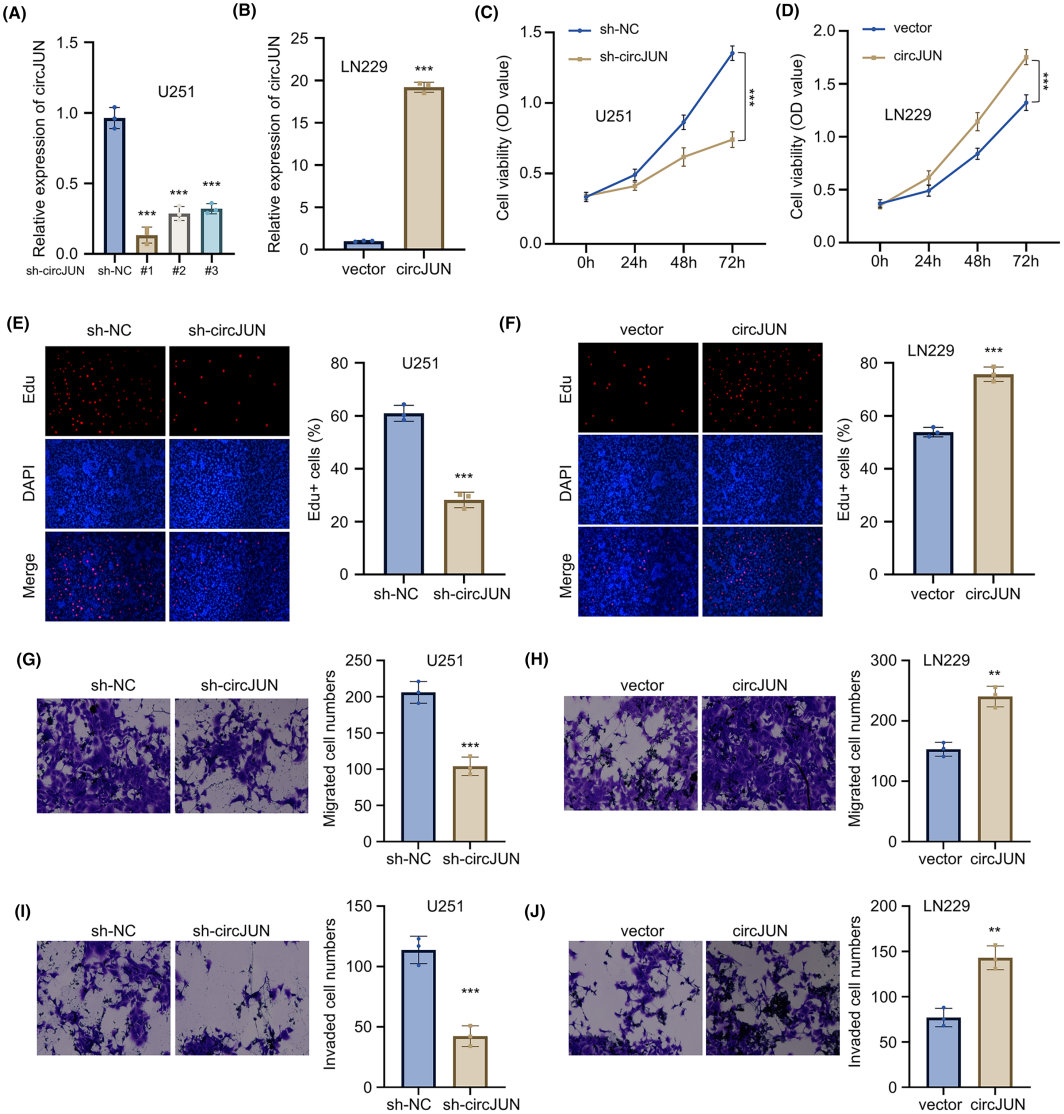
Overexpression of circJUN promotes proliferation and invasion of glioblastoma (GBM) cells. (A) Efficiency of circJUN knockdown in U251 cells using three different shRNAs (sh‐circJUN#1, sh‐circJUN#2 and sh‐circJUN#3), as measured by qRT‐PCR. Sh‐NC served as a negative control. (B) Efficiency of circJUN overexpression in LN229 cells, as determined by qRT‐PCR. Empty vector (EV) served as a control. (C, D) Cell proliferation assessed by CCK‐8 assay in (C) U251 cells with circJUN knockdown and (D) LN229 cells with circJUN overexpression. (E, F) Cell proliferation evaluated by EdU incorporation assay in (E) U251 cells with circJUN knockdown and (F) LN229 cells with circJUN overexpression. (G, H) Cell migration ability assessed by transwell assay without Matrigel in (G) U251 cells with circJUN knockdown and (H) LN229 cells with circJUN overexpression. (I, J) Cell invasion ability evaluated by transwell assay with Matrigel in (I) U251 cells with circJUN knockdown and (J) LN229 cells with circJUN overexpression (*n* = 3, **p < 0.01, ***p < 0.001).

We selected U251 (with the highest level of circJUN expression for knockdown analysis and LN229 (with the lowest expression)) for overexpression analysis through lentivirus‐mediated shRNA or circJUN sequence expression. qRT‐PCR analysis confirmed the downregulation of circJUN after sh‐circJUN expression in U251 cells (Figure 2A) and its overexpression with circJUN coding sequences in LN229 cells (Figure 2B). sh‐circJUN#1 with the strongest silencing effect was used for the subsequent experiments. Knocking down circJUN significantly reduced the proliferation of U251 cells compared to the control group, while overexpression of circJUN enhanced cellular proliferation (Figure 2C,D). The effects on cellular proliferation in both U251 and LN229 cell lines were further confirmed with EdU incorporation assays (Figure 2E,F). Moreover, the migration and invasion capabilities of U251 cells were diminished in cells with circJUN silencing, while circJUN‐overexpressing LN229 cells exhibited increased migration and invasion abilities (Figure 2G–J).

### 3.3 EIF4A3 binds to circJUN to regulate its expression

**FIGURE 3:**
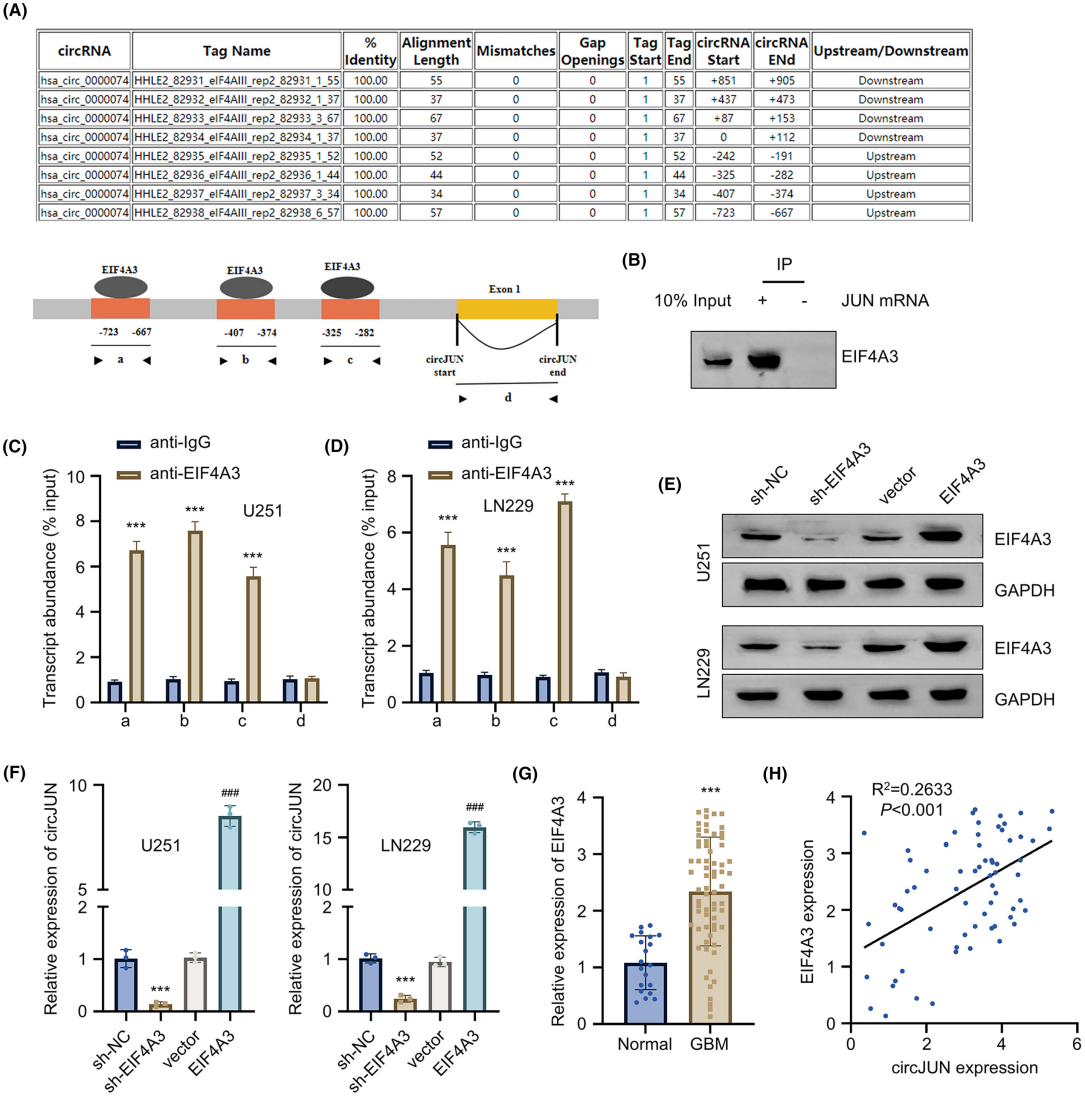
EIF4A3 binds to circJUN to regulate its expression. (A) Predicted EIF4A3 binding sites on circJUN by circinteractome. (B) Western blot showing the precipitation of EIF4A3 protein by circJUN probe in U251 cells. (C, D) qRT‐PCR of 4 predicted binding regions after RNA immunoprecipitation with EIF4A3 antibody in (C) U251 and (D) LN229 cells. (E) EIF4A3 protein levels by Western blot in U251 and LN229 cells with EIF4A3 knockdown or overexpression. (F) CircJUN expression by qRT‐PCR in U251 and LN229 cells with EIF4A3 knockdown or overexpression. (G) EIF4A3 expression in 68 GBM tissues versus 20 normal brain tissues by qRT‐PCR. (H) Correlation between circJUN and EIF4A3 expression in GBM tissues. (*n* = 3 unless especially mentioned in figure panel (*n* = 68 for panel G), **p < 0.01, ***p < 0.001; ^###^ *p* < 0.001 vs. vector).

To identify the regulatory factors of circJUN, we conducted a comprehensive analysis using bioinformatics tools and experimental validation. Through circinteractome prediction, we identified multiple binding sites for EIF4A3 on circJUN sequence (Figure 3A). To confirm this prediction, we divided the sequence containing the binding sites into four regions (a, b, c and d). RNA pull‐down analysis using circJUN probe showed that EIF4A3 protein can be precipitated with circJUN in U251 cells (Figure 3B). Further RIP‐qPCR analysis using EIF4A3 antibody revealed that EIF4A3 could bind to regions a, b and c in both U251 and LN229 cells, but showed no interaction with region d (Figure 3C,D). To elucidate the relationship between EIF4A3 protein and circJUN levels, we performed EIF4A3 knockdown and overexpression studies in U251 and LN229 GBM cell lines (Figure 3E). Silencing of EIF4A3 resulted in decreased circJUN expression compared to the control group, while EIF4A3 overexpression led to increased circJUN levels (Figure 3F). We also observed elevated EIF4A3 levels in GBM tissues compared to healthy controls (Figure 3G). Furthermore, a strong positive correlation was found between EIF4A3 and circJUN expression among GBM cases (Figure 3H). These findings suggest that EIF4A3 plays a crucial role in regulating circJUN expression and may contribute to the pathogenesis of GBM.

### 3.4 Expression of miR‐3064 is regulated by circJUN in GBM cells

**FIGURE 4:**
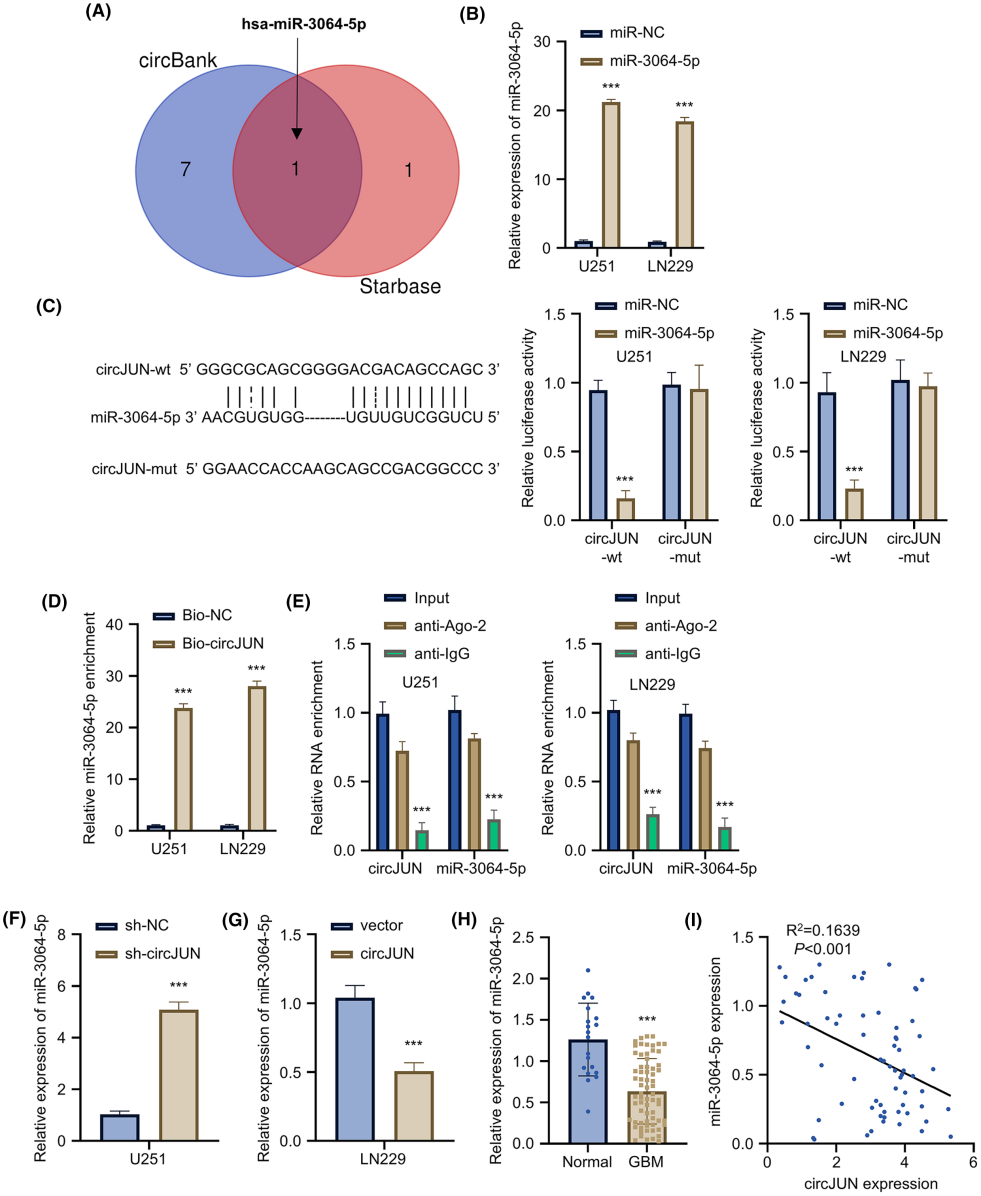
Expression of miR‐3064 is regulated by circJUN in glioblastoma (GBM) cells. (A) Venn diagram of predicted miRNA targets of circJUN by Starbase and circBank. (B) miR‐3064 expression after mimic transfection in U251 and LN229 cells by qRT‐PCR. (C) Luciferase reporter assay with wild‐type or mutant circJUN and miR‐3064 mimic in U251 and LN229 cells. (D) RNA pull‐down with circJUN probe followed by miR‐3064 qRT‐PCR in U251 and LN229 cells. (E) RIP‐qPCR for circJUN and miR‐3064 using anti‐Ago2 antibody in U251 and LN229 cells. (F) miR‐3064 expression after circJUN knockdown in U251 cells by qRT‐PCR. (G) miR‐3064 expression after circJUN overexpression in LN229 cells by qRT‐PCR. (H) miR‐3064 expression in 68 GBM tissues versus 20 normal brain tissues by qRT‐PCR. (I) Correlation between circJUN and miR‐3064 expression in GBM tissues (*n* = 3 unless especially mentioned in figure panel (*n* = 68/20 for panel H), **p < 0.01, ***p < 0.001).

To identify the downstream miRNAs regulated by circJUN, we employed bioinformatics tools starBase and circBank to predict the interacting miRNAs of circJUN. These target predictions revealed miR‐3064 as a common potential target of circJUN (Figure 4A). To study the effects of miR‐3064, we transfected a miR‐3064 mimic into U251 and LN229 GBM cell lines, which significantly increased miR‐3064 levels (Figure 4B). Luciferase reporter assay was performed to validate the binding activity between miR‐3064 and circJUN, using wild‐type (WT) and mutant (mut) circJUN sequences. Transfection of miR‐3064 mimic resulted in reduced luciferase activity in the wild‐type circJUN reporter, while no change was observed in the mutant group (Figure 4C). This decrease in luciferase activity reflected the binding of circJUN to miR‐3064, suggesting a potential regulatory interaction. We further confirmed the interaction between circJUN and miR‐3064 through RNA pull‐down assay, which showed a significantly higher precipitation level of miR‐3064 with circJUN probe compared to the control (Figure 4D). RIP‐qPCR analysis using anti‐Ago2 antibody further demonstrated the association of miR‐3064 and circJUN with Ago‐2 protein (Figure 4E). Overexpression of circJUN led to significant downregulation of miR‐3064 (Figure 4F), while knockdown of circJUN resulted in miR‐3064 upregulation (Figure 4G). In contrast to EIF4A3 and circJUN, miR‐3064 expression was lower in GBM tissue compared to the control group (Figure 4H). Moreover, we observed a significant negative correlation between circJUN and miR‐3064 expression in GBM tissues (Figure 4I). These findings collectively suggest that circJUN may act as a molecular sponge for miR‐3064.

### 3.5 IGFBP5 is a candidate target of miR‐3064 in GBM cells

**FIGURE 5:**
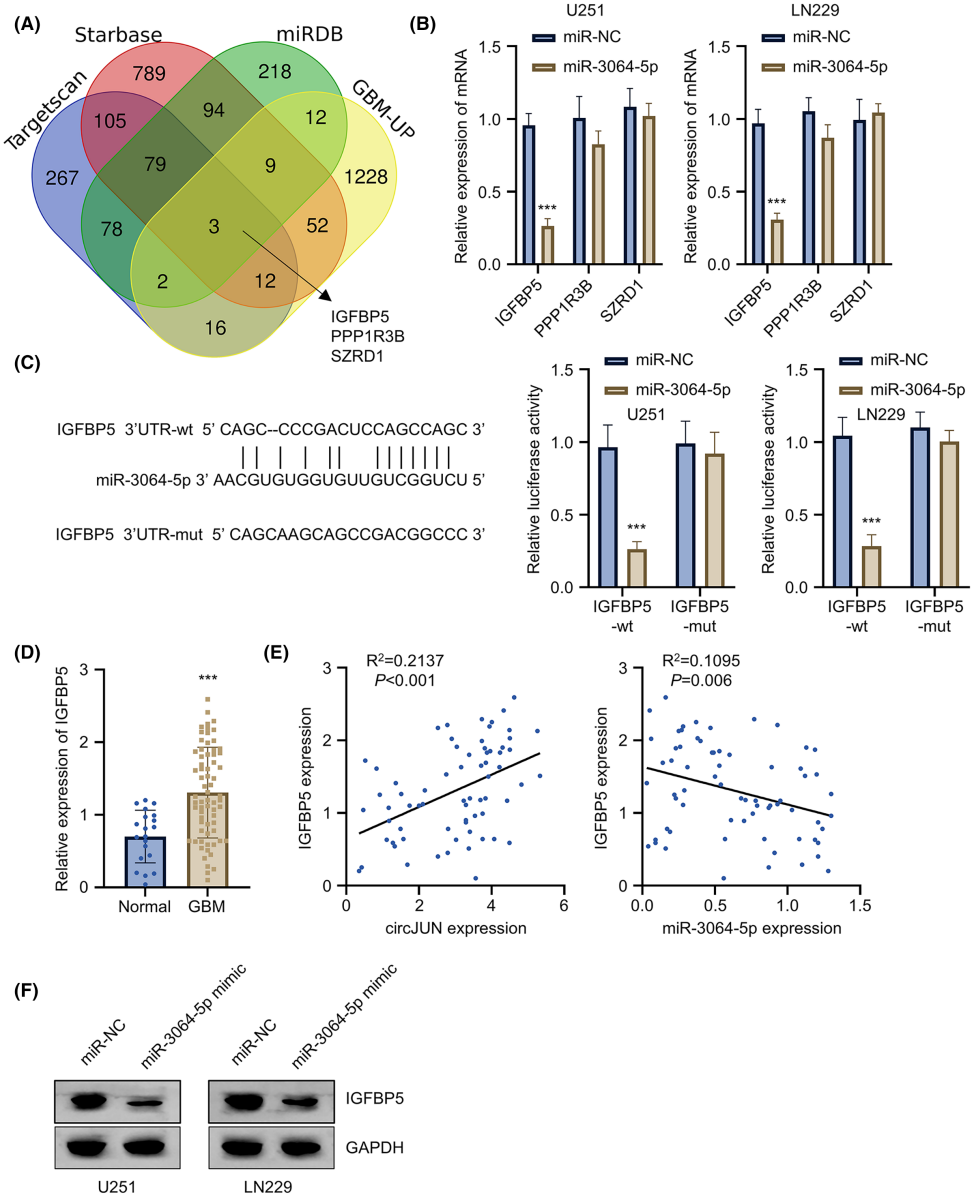
IGFBP5 is a candidate target of miR‐3064 in GBM cells. (A) Venn diagram of predicted miR‐3064 targets by miRDB, Starbase, TargetScan, and upregulated mRNAs in GBM. (B) IGFBP5, PPP1R3B, and SZRD1 expression after miR‐3064 mimic transfection in U251 and LN229 cells by qRT‐PCR. (C) Luciferase reporter assay with wild‐type or mutant IGFBP5 3′UTR and miR‐3064 mimic in U251 and LN229 cells. (D) IGFBP5 expression in 68 GBM tissues versus 20 normal brain tissues by qRT‐PCR. (E) Correlations between circJUN versus IGFBP5 and IGFBP5 versus miR‐3064 expression in GBM tissues. (F) IGFBP5 protein levels after miR‐3064 mimic transfection in U251 and LN229 cells by Western blot. (*n* = 3 unless especially mentioned in figure panel (*n* = 68/20 for panel D), **p < 0.01, ***p < 0.001).

To identify potential gene targets of miR‐3064, we utilized multiple prediction databases (miRDB, Starbase, and TargetScan). The overlap between predicted targets and up‐regulated mRNAs in GBM identified three candidate mRNAs: IGFBP5, PPP1R3B and SZRD1 (Figure 5A). Among these, only IGFBP5 was successfully inhibited by miR‐3064 overexpression in U251 and LN229 GBM cell lines (Figure 5B). To validate the binding activity of miR‐3064 to IGFBP5 mRNA, we conducted luciferase assay using the reporter with wild‐type or mutated binding sites in the presence of miR‐3064 mimic or control miR‐NC. The results confirmed the interaction through predicted binding sites in both U251 and LN229 cell lines (Figure 5C). Similar to the expression patterns of circJUN and EIF4A3, IGFBP5 gene levels were elevated in GBM tissue compared to control samples (Figure 5D). We also observed a positive correlation between circJUN and IGFBP5 expression, while IGFBP5 and miR‐3064 showed a negative correlation (Figure 5E). Consistent with the gene expression correlation between IGFBP5 and miR‐3064, protein expression of IGFBP5 was significantly decreased following transfection with miR‐3064 mimic in both cell lines (Figure 5F). These findings suggest that IGFBP5 is a downstream target of miR‐3064 and may play a role in the circJUN‐miR‐3064 regulatory axis.

### 3.6 The malignancy of GBM cells is regulated by the circJUN‐miR‐3064‐IGFBP5 axis

**FIGURE 6:**
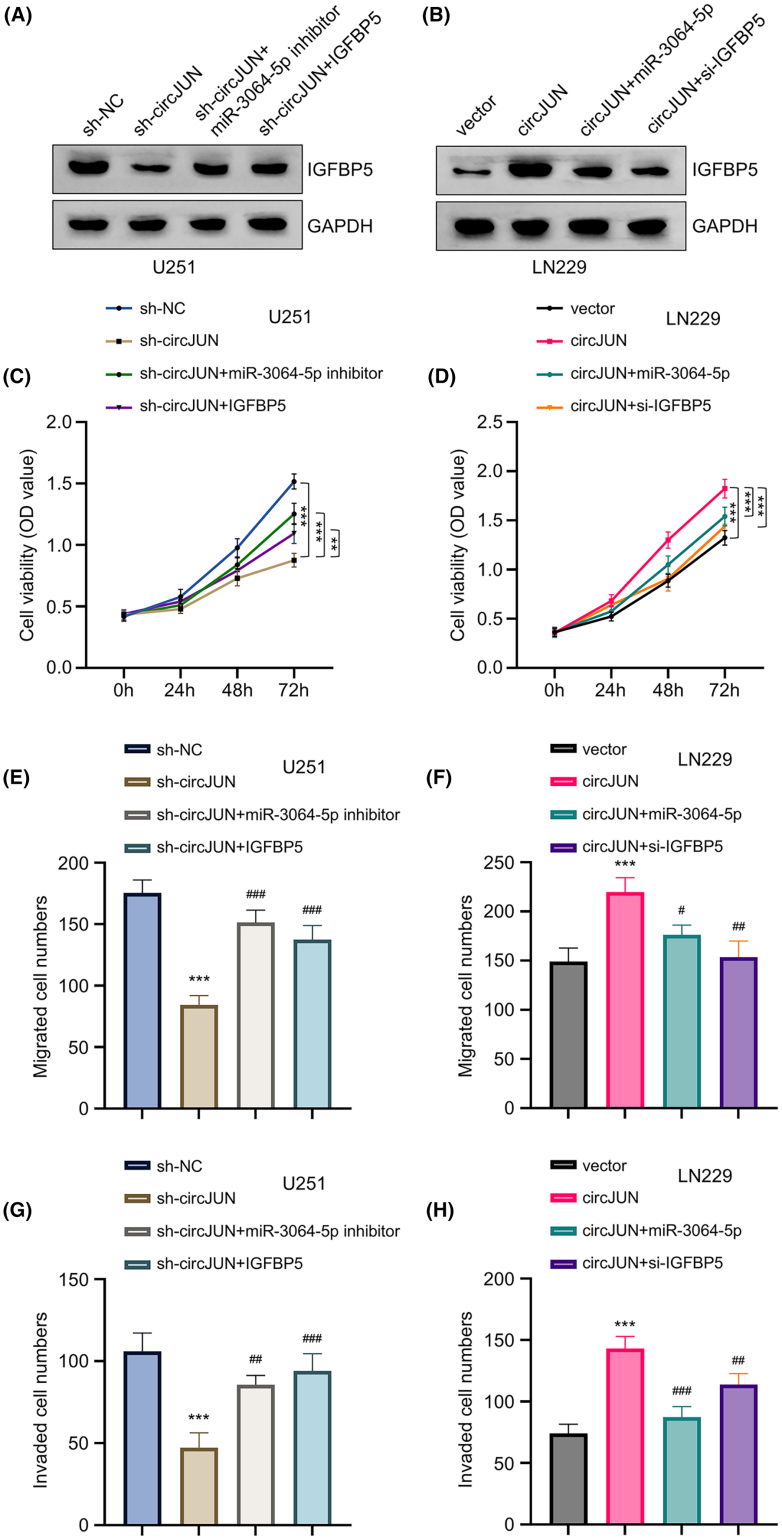
The malignancy of GBM cells is regulated by the circJUN‐miR‐3064‐IGFBP5 axis. (A) IGFBP5 protein levels in U251 cells with circJUN knockdown ± miR‐3064 inhibitor or IGFBP5 overexpression by Western blot. (B) IGFBP5 protein levels in LN229 cells with circJUN overexpression ± miR‐3064 mimic or IGFBP5 siRNA by Western blot. (C, D) Cell proliferation by CCK‐8 assay in (C) U251 and (D) LN229 cells with circJUN modulation ± miR‐3064 or IGFBP5 rescue. (E, F) Cell migration by transwell assay without Matrigel in (E) U251 and (F) LN229 cells with circJUN modulation ± miR‐3064 or IGFBP5 rescue. (G, H) Cell invasion by transwell assay with Matrigel in (G) U251 and (H) LN229 cells with circJUN modulation ± miR‐3064 or IGFBP5 rescue (*n* = 3, **p < 0.01, ***p < 0.001; ^#^ *p* < 0.05 ^##^ *p* < 0.01 ^###^ *p* < 0.001 vs. vector).

We then investigated the functional interplay of circJUN, miR‐3064, and IGFBP5 in regulating cellular behaviours in GBM cell lines. In U251 cells with stable circJUN knockdown, decreased IGFBP5 protein expression was partially rescued by miR‐3064 inhibition or IGFBP5 overexpression (Figure 6A). Conversely, circJUN overexpression in LN229 cells upregulated IGFBP5, while miR‐3064 mimic or IGFBP5 siRNA attenuated this effect (Figure 6B). In U251 cells, circJUN silencing reduced proliferation rates. However, inhibiting miR‐3064 or overexpressing IGFBP5 partially restored proliferation in circJUN‐knockdown cells (Figure 6C). In LN229 cells, circJUN overexpression enhanced proliferation, which was mitigated by miR‐3064 overexpression or IGFBP5 knockdown (Figure 6D). Similar trends were observed in migration and invasion assays (Figure 6E–H). Inhibition of circJUN decreased both migration and invasion capabilities in U251 cells. This effect was partially reversed by miR‐3064 inhibition or IGFBP5 overexpression (Figure 6E,G). In contrast, circJUN overexpression enhanced migration and invasion of LN229 cells, while miR‐3064 overexpression or IGFBP5 inhibition attenuated these effects (Figure 6F,H). These findings collectively suggest that the circJUN‐miR‐3064‐IGFBP5 axis plays a crucial role in regulating proliferation, migration and invasion of GBM cells, potentially contributing to the aggressive nature of this malignancy.

### 3.7 CircJUN promotes tumour growth of GBM cells in a nude mouse xenograft model

**FIGURE 7:**
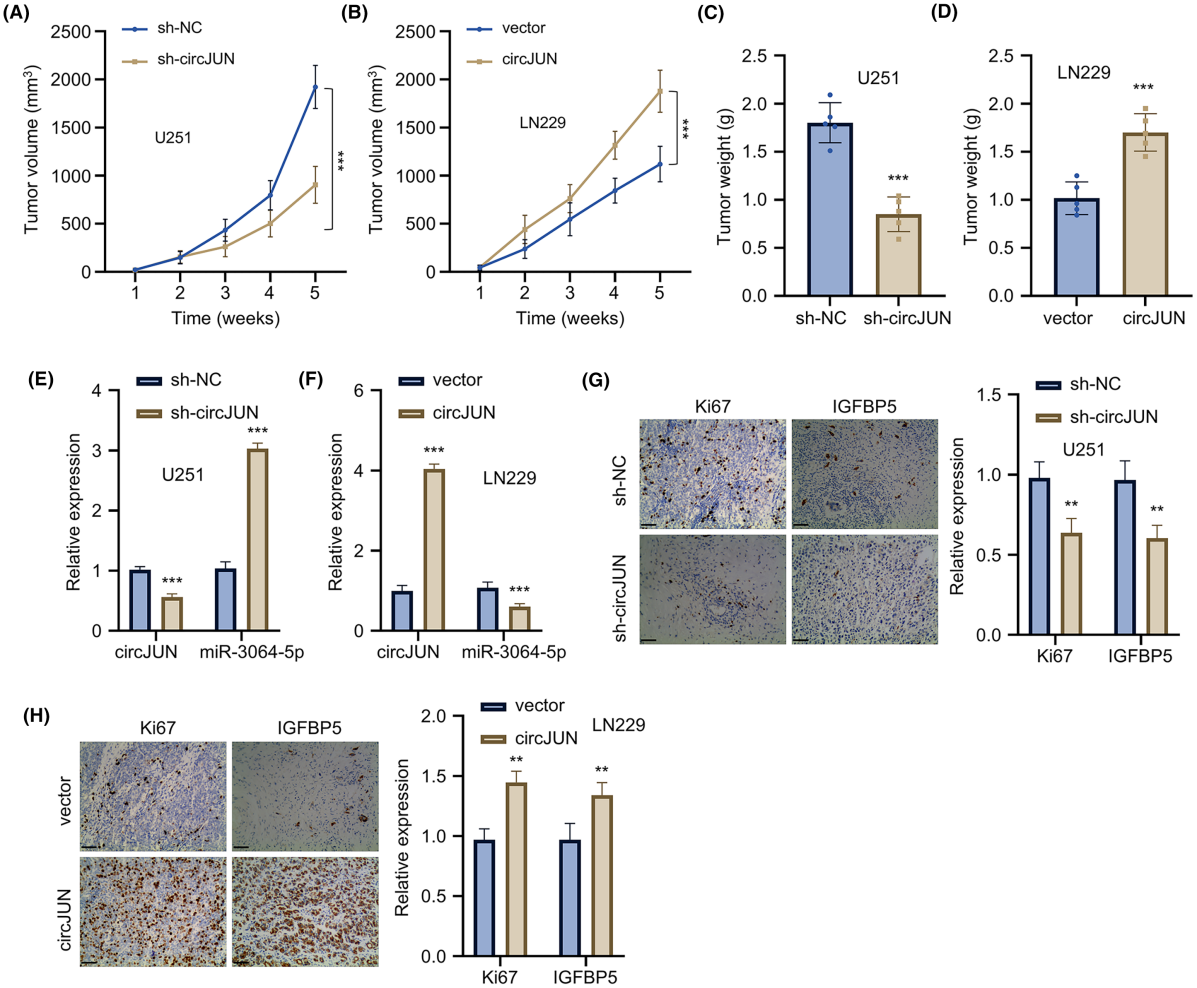
CircJUN promotes tumour growth of GBM cells in a nude mouse xenograft model. (A). Tumour volume growth curves of U251 xenografts with circJUN knockdown in nude mice (*n* = 5 per group). (B) Tumour volume growth curves of LN229 xenografts with circJUN overexpression in nude mice (*n* = 5 per group). (C, D) Tumour weights at sacrifice for (C) U251 and (D) LN229 xenografts. (E, F) CircJUN and miR‐3064 expression in tumour tissues from (E) U251 and (F) LN229 xenografts by qRT‐PCR. (G, H). Ki67 and IGFBP5 protein expression levels in tumour tissues from (G) U251 and (H) LN229 xenografts analysed by IHC staining (*n* = 5 mice per group, **p < 0.01, ***p < 0.001).

To investigate the role of circJUN in tumour formation in vivo, we conducted xenograft experiments using engineered U251 cells with sh‐circJUN expression and LN229 cells with circJUN overexpression. Knockdown of circJUN in U251 cells significantly slowed tumour formation in nude mice (Figure 7A), while overexpression of circJUN in LN229 cells promoted tumour growth (Figure 7B). Consistent with the tumour volume changes, tumour weight was lower in the circJUN‐knockdown U251 group (Figure 7C), and higher in the circJUN‐overexpressing LN229 group (Figure 7D). Gene expression analysis of the tumour tissues revealed that circJUN silencing in U251‐derived tumours led to upregulation of miR‐3064 (Figure 7E), while circJUN overexpression in LN229‐derived tumours resulted in lower miR‐3064 expression (Figure 7F). IHC analysis of tumour tissues showed lower levels of IGFBP5 and Ki67 proteins in circJUN‐silenced tumours (Figure 7G), while tumours from circJUN‐overexpressing tumours showed increased protein levels of both IGFBP5 and Ki67 (Figure 7H). These in vivo findings further support the critical role of the circJUN‐miR‐3064‐IGFBP5 axis in GBM progression.

## 4 DISCUSSION

Glioblastoma (GBM), a WHO grade IV glioma, is one of the most prevalent and aggressive brain tumours.^2^, ^15^ Researchers have investigated various molecular mechanisms and identified several pathways potentially regulating GBM invasion and migration.^16^, ^17^, ^18^, ^19^, ^20^ Among different molecular pathways, miRNA‐mediated pathways have been extensively studied, and multiple miRNAs have been identified that can serve as either biomarkers or potential therapeutic agents against GBM,^17^, ^19^ such as miR‐5096, miR‐10b, and miR‐709.^21^, ^22^, ^23^ Due to the nature of miRNA interactions in transcription and translation, one or more specific downstream pathways involving other mRNAs and protein translations play central roles in regulating GBM pathogenesis. In our study, we have elucidated a novel mechanism: circJUN functions as an oncogene, regulating GBM proliferation and invasion through the miR‐3064‐IGFBP5 axis.

Our results demonstrate that circJUN plays a key role in promoting GBM progression. We identified circJUN through analysis of large datasets containing various patient samples and confirmed its higher expression in GBM tumours compared to healthy controls using qRT‐PCR. Through various experimental models, we demonstrated that knockdown of circJUN inhibits GBM proliferation and invasion, suggesting its potential as a therapeutic target for GBM treatment. Previous studies have shown that c‐JUN accumulates in GBM and is associated with cellular proliferation.^24^ As a circular structure derived from c‐JUN, circJUN exhibits enhanced stability and a longer half‐life compared to its linear counterpart. Our findings align with previous research suggesting that inhibition of c‐JUN‐related molecules could serve as a potential therapeutic approach in GBM treatment.^24^

Our study revealed that circJUN interacts with miR‐3064, a miRNA previously identified as an oncogene in pancreatic cancer^25^ and a tumour suppressor in ovarian cancer.^26^ In the context of GBM, we demonstrated that miR‐3064 expression is suppressed by circJUN. Notably, lower expression levels of miR‐3064 were observed in GBM tumours compared to healthy controls, which inversely correlates with circJUN expression. The interaction between circJUN and miR‐3064 represents a novel regulatory axis in GBM. By acting as a molecular sponge, circJUN sequesters and represses miR‐3064 activity, preventing it from binding to its downstream targets. This mechanism allows circJUN to indirectly promote the expression of miR‐3064 targets, thereby contributing to GBM progression. Nevertheless, the exact mechanisms by which circJUN interaction impacts on miR‐3064 stability require further investigation. In addition, while our study focused on the circJUN‐miR‐3064 interaction, other miRNAs such as miR‐125b have been shown to inhibit c‐JUN and suppress melanoma progression.^27^ Further research is needed to explore additional miRNA targets of circJUN and their potential roles in GBM pathogenesis. Understanding these complex regulatory networks could lead to the development of more effective therapeutic strategies for controlling GBM.

In addition to its interaction with circJUN, we demonstrated that miR‐3064 regulates the expression of IGFBP5, a protein known to promote GBM invasion through EMT and AKT signalling.^28^ Our results confirmed higher expression of IGFBP5 in GBM patients compared to healthy controls, consistent with previous findings.^29^ Interestingly, different types of IGFBPs can play opposing roles in GBM regulation, as exemplified by IGFBP3, which functions differently from IGFBP5.^30^ Our experiments revealed that overexpression of miR‐3064 decreased IGFBP5 levels, leading to impaired cellular proliferation, migration and invasion in GBM cell lines. This indicates that miR‐3064 inhibits GBM invasion and proliferation by regulating IGFBP5. These findings underscore the role of miR‐3064 as a tumour suppressor in GBM, exerting its anti‐tumorigenic effects through the circJUN‐miR‐3064‐IGFBP5 axis.

To conclude, this study unveils a novel regulatory axis in GBM pathogenesis involving circJUN, miR‐3064, and IGFBP5. CircJUN, significantly overexpressed in GBM tissues and correlated with poor patient prognosis, promotes tumour cell proliferation and invasion. Mechanistically, EIF4A3 interacts with and enhances circJUN expression, while circJUN acts as a molecular sponge for miR‐3064, thereby regulating IGFBP5 expression and modulating GBM cell malignancy. These findings provide new insights into GBM progression and identify potential therapeutic targets and biomarkers. Future research should focus on exploring circJUN as a diagnostic biomarker, and investigating its interplay with other GBM‐associated pathways to develop more comprehensive treatment strategies for this aggressive brain tumour.

## AUTHOR CONTRIBUTIONS

**Yuhao Zhang:** Conceptualization (equal); data curation (equal); investigation (equal); methodology (equal); writing – original draft (equal). **Shiming Liu:** Conceptualization (equal); investigation (equal); methodology (equal); writing – original draft (equal). **Cheng Wu:** Data curation (equal); investigation (equal); writing – original draft (equal). **Xin Gao:** Investigation (equal); methodology (equal). **Hongtao Zhao:** Investigation (equal); methodology (equal); writing – original draft (equal). **Ou Li:** Investigation (equal). **Faliang Gao:** Conceptualization (equal); funding acquisition (equal); methodology (equal); writing – original draft (equal); writing – review and editing (equal).

## FUNDING INFORMATION

China Postdoctoral Science Foundation (approval no. 2023M733164).

## CONFLICT OF INTEREST STATEMENT

The authors declare that they have no conflict of interest.

## Data Availability

Not applicable.
